# The Risk of Adverse Effects of TNF-α Inhibitors in Patients With Rheumatoid Arthritis: A Network Meta-Analysis

**DOI:** 10.3389/fimmu.2022.814429

**Published:** 2022-02-16

**Authors:** Bei He, Yun Li, Wen-wen Luo, Xuan Cheng, Huai-rong Xiang, Qi-zhi Zhang, Jie He, Wen-xing Peng

**Affiliations:** ^1^ Department of Pharmacy, The Second Xiangya Hospital, Central South University, Changsha, China; ^2^ Institute of Clinical Pharmacy, Central South University, Changsha, China

**Keywords:** adverse effects, TNF-α inhibitors, rheumatoid arthritis, network meta-analysis, serious adverse events

## Abstract

**Objectives:**

To evaluate the safety of each anti-TNF therapy for patients with rheumatoid arthritis (RA) and then make the best choice in clinical practice.

**Methods:**

We searched PUBMED, EMBASE, and the Cochrane Library. The deadline for retrieval is August 2021. The ORs, Confidence Intervals (CIs), and p values were calculated by STATA.16.0 software for assessment.

**Result:**

72 RCTs involving 28332 subjects were included. AEs were more common with adalimumab combined disease-modifying anti-rheumatic drugs (DMARDs) compared with placebo (OR = 1.60, 95% CI: 1.06, 2.42), DMARDs (1.28, 95% CI: 1.08, 1.52), etanercept combined DMARDs (1.32, 95% CI: 1.03, 1.67); certolizumab combined DMARDs compared with placebo (1.63, 95% CI: 1.07, 2.46), DMARDs (1.30, 95% CI: 1.10, 1.54), etanercept combined DMARDs (1.34, 95% CI: 1.05, 1.70). In SAEs, comparisons between treatments showed adalimumab (0.20, 95% CI: 0.07, 0.59), etanercept combined DMARDs (0.39, 95% CI: 0.15, 0.96), golimumab (0.19, 95% CI: 0.05, 0.77), infliximab (0.15, 95% CI: 0.03,0.71) decreased the risk of SAEs compared with golimumab combined DMARDs. In infections, comparisons between treatments showed adalimumab combined DMARDs (0.59, 95% CI: 0.37, 0.95), etanercept (0.49, 95% CI: 0.28, 0.88), etanercept combined DMARDs (0.56, 95% CI: 0.35, 0.91), golimumab combined DMARDs (0.51, 95% CI: 0.31, 0.83) decreased the risk of infections compared with infliximab combined DMARDs. No evidence indicated that the use of TNF-α inhibitors influenced the risk of serious infections, malignant tumors.

**Conclusion:**

In conclusion, we regard etanercept monotherapy as the optimal choice for RA patients in clinical practice when the efficacy is similar. Conversely, certolizumab + DMARDs therapy is not recommended.

**Systematic Review Registration:**

identifier PROSPERO CRD42021276176.

## Introduction

Rheumatoid arthritis (RA) is one of the most prevalent chronic inflammatory diseases, which can cause cartilage and bone damage as well as a disability that carries a substantial burden for both the individual and society (1). Currently, antitumors necrosis factor (anti-TNF) therapy has been established as an efficacious therapeutic strategy in RA (2). TNF-α is a pro-inflammatory cytokine known to have a key role in the pathogenesis of chronic immune-mediated diseases (3). Five TNF-α inhibitors have received regulatory approval for clinical use in rheumatology: adalimumab, golimumab, infliximab, certolizumab, and etanercept (4). They are commonly used in the treatment of rheumatoid arthritis.

Besides therapeutic effects, some studies reported that TNF-α inhibitors may also cause some adverse effects in patients with RA (5–8). Although there have been some pair-wise meta-analyses and network meta-analyses that evaluate the safety of different TNF-α inhibitors therapies for patients with RA. Nevertheless, most of the trials only focused on total AEs and SAEs or just one kind of detailed AEs, and some of the initial meta-analyses were contradicted by subsequent studies. For instance, Bongartz et al. reported that RA patients who were treated by anti-TNF therapies had an increased risk of serious infections and malignancies (9), while another trial evaluating malignancy risk in RA patients concluded that there was no significant evidence of an increased risk of malignancy using TNF-α inhibitors (10).

To evaluate the safety of TNF-α inhibitors in patients with RA, we choose six safety outcomes to systematically assess 10 anti-TNF therapies from 72 RCTs with a sample size of 28332 patients. Our network meta-analysis seeks to infer the risk of adverse effects of two therapies in patients with rheumatoid arthritis by direct and indirect comparisons. Simultaneously, it extracts and analyzes data from all randomized control trials (RCTs) to select the best therapy. The objective of the current study is to better characterize the safety of each anti-TNF therapy for patients with RA and then make the best choice in clinical practice.

## Method

### Study Selection

We searched PUBMED, EMBASE, and the Cochrane Library with the terms of drugs (adalimumab, certolizumab, etanercept, infliximab, and golimumab) and diseases (rheumatoid arthritis). After matching each “drug” and “disease”, restricting search results with the condition “randomized controlled trial”, we finally form the retrieval expressions that adapt to different databases. The deadline for retrieval is August 2021. Two investigators performed the literature screening according to the inclusion and exclusion criteria independently. The repeated studies were excluded firstly. Afterward, excluded unrelated studies by reading the titles and abstracts. The literature that met the inclusion and exclusion criteria was further screened by reading the full text. Disagreements were resolved by consensus Equations.

### Inclusive Criteria

RCTs associated with adalimumab, certolizumab, etanercept, infliximab, and golimumab in the treatment of rheumatic diseases are included. Subjects should be greater than or equal to 18 years old and should be diagnosed with rheumatoid arthritis according to American College of Rheumatology criteria or other authoritative criteria. Disease progression, race, nationality, and complications are not limited. For the types of interventions, the experimental groups use TNF-α inhibitors, with or without disease-modifying antirheumatic drugs (DMARDs). The control groups use placebo (with or without DMARDs) or DMARDs alone.

### Exclusive Criteria

RCTs that accord with any of the following criteria will be excluded: (1) studies with no accessible records of AE, SAE, malignant tumors, infections, severe infections, or malignant tumors (requiring intravenous antibiotic treatment or hospitalization or threatening patient’s life); (2) repetitive studies with shorter follow-up time; (3) studies with improper control (other therapy in experimental group or control group); (4) studies with Jadad score lower than or equal to 3 points; (5) studies with full texts not available.

### Data Extraction

Data extraction was performed independently by He Bei and Li Yun, and the EndNote software was used to filter duplications and irrelevant literature by reading titles and abstracts. The remaining articles were then browsed in full text to determine whether they met the inclusion criteria. After removing ineligible publications, the two reviewers independently extracted data from each study, and disagreements were resolved by reaching a consensus. From each eligible study, we extracted and summarized the following details: the first author, year of publication, country, the total number of participants, type of TNF-α inhibitors, age range, follow-up time, duration of trials.

### Assessment of Risk of Bias

Two investigators independently assessed each study’s risk of bias as low, unclear, and high. Disagreements were resolved by consensus. The items included: Random sequence generation; allocation concealment; blinding of participants and personnel; blinding of outcome assessment; incomplete outcome data; selective reporting; other bias.

### Quality Assessment

Two reviewers independently used the modified Jadad scale to assess the quality of RCTs (randomized control trials). NOS includes three aspects (selection, comparability, and exposure for case-control studies or outcomes for cohort studies), as well as scores of 4, 2, and 3, respectively. The modified Jadad scale comprises four parts: generation of the allocation sequence, concealment of allocation, blinding, and incomplete outcome data, and scores of 2, 2, 2, and 1 for four parts, respectively. Studies with scores of 1-3 were considered to be of low quality; 4-7 high quality.

### Data Synthesis and Analysis

Network meta-analysis was performed to compare each of the 10 anti-TNF therapies. Based on the multivariate framework, the network meta-analysis was conducted using frequency theory, and two program packages, network, and mvmeta, developed by STATA 16 software based on multiple regression theory, were used for statistical analysis. Firstly, an evidence network diagram was drawn to show the comparison between interventions, and the consistency test was conducted according to the existence of closed rings. Second, for counting data, OR was used for calculation, the network meta of adverse drug reactions was analyzed, 95% confidence interval was used for all effect sizes, and 95%CI of OR did not cross effect line 1, indicating that P<0.05 was statistically significant. SUCRA analysis was used to seek therapies that had the highest probability of adverse events, with the higher the SUCRA value, the higher the risk. Stata 16.0 draws a comparative-correction funnel plot to determine whether there is a small sample effect in the analysis and recognition network, to evaluate the publication bias of the final screening. All tests were two-sided with a significance level of 0.05.

## Result

**Figure 1:**
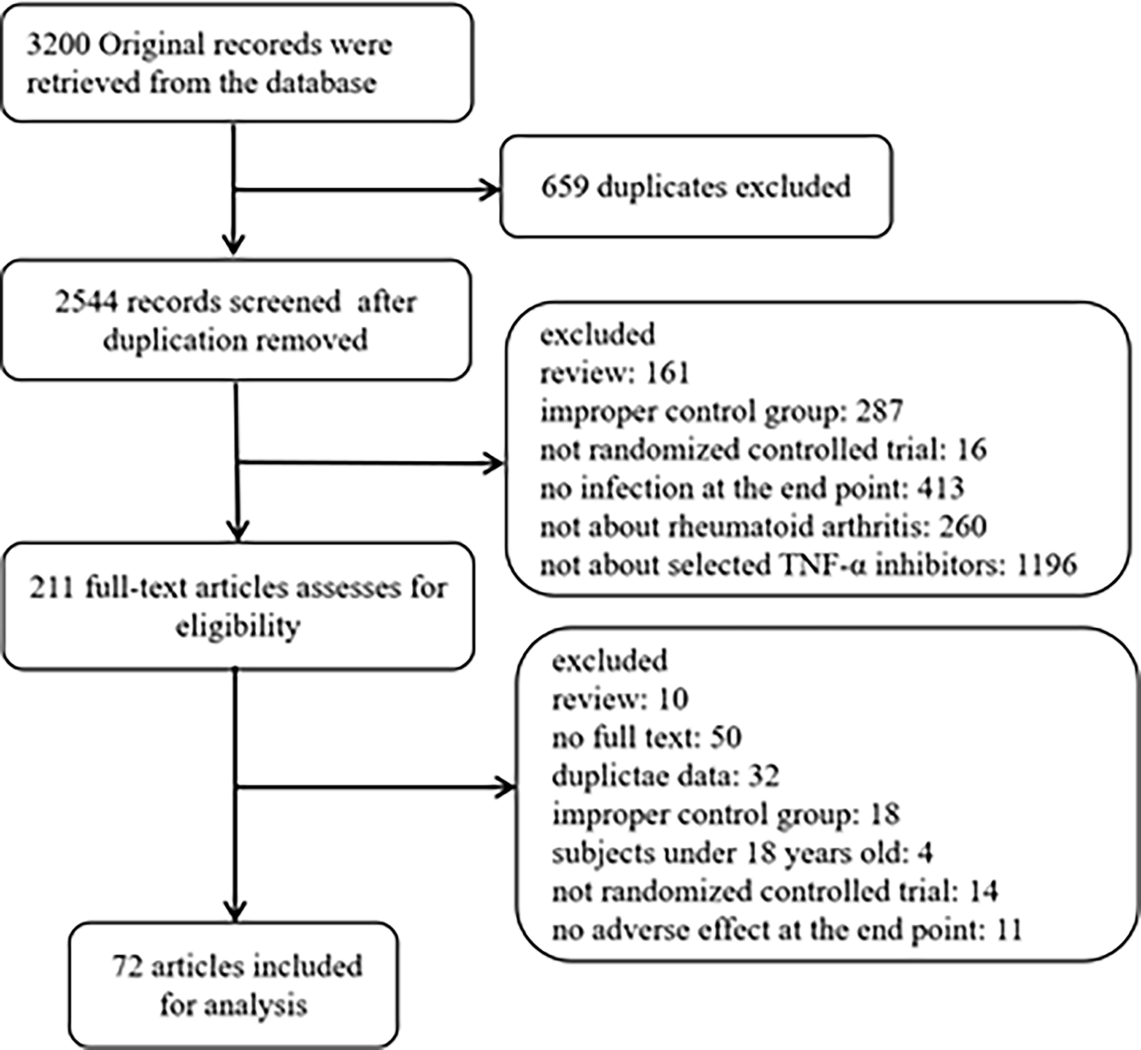
Flow diagram of search results.

By searching databases, we retrieved 3200 original records. After excluding duplicates and irrelevant articles, 211 full-text articles were assessed for eligibility. By reading full-text, 72 articles met the inclusive criteria and exclusive criteria (11–82). The following diagram of the study selection process for this meta-analysis is shown in **Figure 1**. The 72 articles included 28332 patients, followed up for about 16-104 weeks. 72 articles involved RCT experiments, including 21 adalimumab trials, 13 certolizumab trials, 21 etanercept trials, 9 golimumab trials, and 8 infliximab trials. **Table 1** summarizes the relevant characteristics.

**Table 1:** Characteristics of included studies.

Author, Year		Duration of trials (years)	Quality score	Follow-up time(Week)	Average age(years old)	Duration of rheumatoid arthritis (years)	Number of women(n)	Number of patients (n)	Total number of cases (n)	Intervention measures
Den et al. (11)		NA	4	4	55	11.9	22	31	120	Placebo
					54	11	10	17		adalimumab 0.5mg/Kg
					58	11.2	10	18		adalimumab 1mg/Kg
					54	10.8	8	18		adalimumab 3mg/Kg
					59	14.5	15	18		adalimumab 5mg/Kg
					53	8.9	12	18		adalimumab 10mg/Kg
Frust et al. (15)		NA	4	24	55	9.3	253	318	636	adalimumab 40mg eow+DMARD
					55.8	11.5	252	318		placebo+DMARD
Van der Putte et al. (13)		NA	4	12	53.7	10.4	61	72	284	adalimumab 20mg qw
					52.6	10	57	70		adalimumab 40mg qw
					53.2	10.1	50	72		adalimumab 80mg qw
					50.2	9.4	57	70		placebo
Weinblatt et al. (14)		NA	5	24	53.5	13.1	52	69	271	adalimumab 20mg eow+MTX
					57.2	12.2	50	67		adalimumab 40mg eow+MTX
					55.5	12.8	55	73		adalimumab 80mg eow+MTX
					56	11.1	51	62		placebo+MTX
Keystone et al. (16)		NA	5	52	56.1	11	158	207	619	adalimumab 40mg eow+MTX
					57.3	11	160	212		adalimumab 20mg qw+MTX
					56.3	10.9	146	200		placebo+MTX
van der Putte et al. (19)		2000.1-2001.6	7	26	53.1	9.3	84	106	544	adalimumab 20mg eow
					54.4	11.3	81	112		adalimumab 20mg qw
					52.7	10.6	90	113		adalimumab 40mg eow
					51.8	11.9	81	103		adalimumab 40mg qw
					53.5	11.6	85	110		placebo
Breedveld et al. (20)	PREMIER(NCT00195663)	NA	6	104	51.9	0.7	193	268	799	adalimumab 40mg eow+MTX
					52.1	0.7	212	274		adalimumab 40mg eow
					52	0.8	190	257		placebo+MTX
Kim et al. (25)		NA	5	18	48.5	6.8	62	65	128	40 mg adalimumab eow+MTX
					49.8	6.9	53	63		placebo +MTX
Miyasaka et al. (31)	CHANGE	2004.2-2005.6	5	24	54.8	10	69	87	352	adalimumab 20mg eow
					56.9	9.9	72	91		adalimumab 40mg eow
					54.3	9.5	72	87		adalimumab 80mg eow
					53.4	8.4	67	87		placebo
Bejarano et al. (28)		2003.3.5-2004.12.2	7	56	47	9.5	44	75	148	adalimumab 40mg eow+MTX
					47	7.9	39	73		placebo+MTX
Chen et al. (33)		NA	5	12	53	6.2	26	35	47	adalimumab 40mg eow+MTX
					53	8.3	11	12		MTX
van Vollenhoven et al. (46)	NCT00853385	2009.1.30-2011.2.10	3	12	52.5	8.1	162	204	312	adalimumab 40 mg eow
					53.7	7.9	82	108		placebo
Detert et al. (48)	HIT HARD	2007.6-2010.9	5	24	47.2	0.15	61	87	172	adalimumab 40mg eow+MTX
					52.5	0.14	57	85		MTX
Kavanaugh et al. (49)	OPTIMA(NCT00420927)	2006.12-2010.7	5	26	50.7	0.33	380	515	1032	adalimumab 40mg eow+MTX
					50.4	0.38	382	517		placebo+MTX
Hørslev-Petersen et al. (57)	OPERA	2007.8-2009.12	5	104	56.2	0.24	56	89	180	adalimumab 40mg eow+MTX
					54.2	0.23	63	91		placebo+MTX
Kennedy et al. (58)	ALTARA	2010.11-2012.7	5	12	50.2	NR	78	85	214	patecilizumab
					50.6	NR	68	85		adalimumab 40mg eow
					48.8	NR	37	44		placebo
Takeuchi et al. (62)	HOPEFUL 1	2009.3-2010.11	5	26	54	0.3	144	171	334	adalimumab 40mg eow+MTX
					54	0.3	128	163		placebo+MTX
Taylor et al. (74)	RA-BEAM	2012.11-2015.9	5	24	53	10	251	330	818	adalimumab 40mg eow
					53	10	382	488		placebo
Fleischmann et al. (77)	SELECT - COMPARE	2015.12-2017.6	6	26	54	8	512	651	1629	placebo +MTX
					54	8	159	327		adalimumab 40 mg+MTX
Ducourau et al. (78)	(NCT01895764)	2013.3-2014.10	4	26	43	3	22	52	107	adalimumab 40mg qw+MTX
					41	2	28	55		adalimumab 40 mg qw
Combe et al. (81)	NCT02889796	2016.8.30-2019.6.20	7	24	53	8	266	325	800	adalimumab 40 mg biw+MTX
					53	7.3	391	475		placebo +MTX
Fleischman et al. (77)	FAST4WARD	2003.6-2004.7	6	24	52.7	8.7	87	111	220	certolizumab 400mg
					54.9	10.4	97	109		placebo
Smolen et al., 2009	RAPID 2	20005.6-2006.9	4	24	51.9	6.5	192	246	619	certolizumab 400mg + MTX
					52.2	6.1	206	248		certolizumab 200mg + MTX
					51.5	5.6	107	125		placebo + MTX
Choy et al. (42)	NCT00544154	2002.10-2004.1	7	24	53	9.4	91	126	247	certolizumab 400mg + MTX
					55.6	9.9	80	121		placebo + MTX
Weinblatt et al. (47)	REALISTIC(NCT00717236)	2008.7-2010.3	7	12	55.4	8.6	660	q	1063	certolizumab (certolizumab 400 mg qw 0, 2 and 4,followed by certolizumab 200 mg eow)+DMARDs
					53.9	8.9	169	212		placebo +DMARDs
schiff et al. (61)	NCT01147341		4	52	56.1	12	NR	27	37	certolizumab(400 mg qw 0, 2 and 4, followed by 200mg eow)+DMARDs
					59	14	NR	10		placebo +DMARDs
Yamamoto et al. (63)	J-RAPID	2008.11.19-2010.8.18	7	24	54.3	6.0	58	72	316	certolizumab 100mg eow + MTX
					50.6	5.6	69	82		certolizumab 200mg eow + MTX
					55.4	6.0	69	85		certolizumab 400mg eow + MTX
					51.9	5.8	66	77		placebo + MTX
Furst et al. (64)	DOSEFLEX		5	16	51.5	6.5	56	69	208	Placebo +MTX
					55.6	5.9	49	70		certolizumab 200 mg eow +MTX
					53.1	6.4	57	69		certolizumab 400 mg q4w +MTX
Smolen et al. (65)	CERTAIN	2008.6-2010.12	5	24	53.6	4.5	81	96	194	certolizumab(400 mg certolizumab qw 0, 2 and 4, followed by 200 mg certolizumab eow)+DMARDs
					54	4.7	75	98		placebo +DMARDs
Atsumi et al. (66)	C-OPERA (NCT01451203)	2011.10-2013.8	7	52	49.4	4.0	129	159	316	certolizumab 400mg/200mg eow +MTX
					49	4.3	127	157		placebo + MTX
Emery et al. (72)	C-EARLY (NCT01519791)	2012.1-2015.9	6	52	50.4	0.24	497	660	879	certolizumab 400mg/200mg eow +MTX
					51.2	0.24	170	219		placebo + MTX
Kang et al. (75)	(NCT00993317)	2009.12-2011.8	4	24	51.6	6.5	72	85	127	certolizumab 400mg/200mg eow +MTX
					50.8	5.5	35	42		placebo + MTX
Bi et al. (76)	RAPID-C (NCT02151851)	2014.7.23-2016.6.17	6	24	48.2	7.0	268	316	429	certolizumab 200 mg eow (loading dose: 400 mg certolizumab qw 0, 2 and 4) + MTX
					47.1	6.6	95	113		(PBO) + MTX
Hetland et al. (79)	NCT01491815	2012.12.3-2018.12.11	6	24	54.6	0.53	139	197	399	active conventional treatment
					55.3	0.56	139	202		certolizumab 200 mg qw (400 mg qw 0, 2, and 4)+MTX
Genovese et al. (39)		1997.5-1999.3	5	104	49	1	75	217	632	three 2.5-mg MTX qw and placebo biw
					50	0.9	75	208		10 mg of etanercept biw and three placebo tablets qw,
					51	1	74	207		25 mg of etanercept biw and three placebo tablets qw
Smolen et al. (1)		2011.12.14-2013.11.11	4	12	53	5.9	96	457	914	certolizumab pegol (400 mg weeks 0, 2,
								457		adalimumab (40 mg once q2w) plus
Keystone et al. (16)		NA	5	8	54	10.8	38	53	420	placebo
					53	9.0	169	214		50 mg etanercept qw
					52	8.2	121	153		25 mg etanercept biw
van der Heijde et al. (26)	TEMPO	2000.10-2001.7	6	104	52.5	6·8	171	231	682	etanercept 25mg biw + MTX
					53.2	6·8	171	223		etanercept 25mg biw + placebo
					53	6·3	180	228		placebo + MTX
Lan et al. (21)		NR	4	12	47.55	NR	50	29	58	etanercept 25mg biw + MTX
					50.79			29		placebo +MTX
van Riel et al. (22)	ADORE	2003.3-2004.5	4	16	53	10	126	159	314	etanercept 25 mg biw
					54	9.8	119	155		etanercept 25 mg biw + MTX
Weisman et al. (27)	RA	NA	6	16	60.6	10.1	192	266	535	etanercept 25mg biw
					59.3	9.4	210	269		placebo
Emery et al. (29)	COMET	2004.10-2006.2	7	52	50.5	8·8	196	274	542	etanercept 50mg qw + MTX
					52.3	9·3	191	268		MTX
Kameda et al. (41)	JESMR(NCT00688103)	2005.6-2007.1	4	24	58.1	10.6	62	71	146	etanercept 25 mg eow
					56.5	8.1	60	75		MTX+etanercept
Jobanputra et al. (43)	EU Clinical	2007.5-2010.4	4	52	55	7.0	15	60	120	adalimumab 40 mg qw
	Trials Register 2006-006275-21/GB				53.2	5.5	18	60		etanercept 50 mg qw
Kim et al. (44)	APPEAL	2007.6-2009.3	6	16	48.4	6.5	17	197	300	etanercept 25 mg biw+MTX
					48.5	6.9	12	103		DMARD+MTX
Takeuchi et al. (80)	NCT00445770	NA	6	52	51.8	3.0	145	182	550	etanercept 25 mg biw
					51.5	2.9	154	192		etanercept 10 mg biw
					50.4	3.0	140	176		MTX
Emery et al. (56)	NCT00913458	2009.10.20-2012.12.17	5	39	49.6	0.54	47	63	193	etanercept (25 mg)+MTX
					47.7	0.58	36	65		placebo +MTX
					50.9	0.59	42	65		placebo
Machado et al. (59)	NCT00848354	2009.6-2011.3	5	24	48.4	7.9	248	281	423	etanercept(50 mg qw)+MTX
					48.6	9.0	128	142		(DMARD) + MTX
Nam et al. (60)	EMPIRE	2006.10-2009.5	7	78	47.9	0.5	44	55	110	etanercept 50mg qw + MTX
					48.4	0.67	40	55		placebo + MTX
Smolen et al. (52)	PRESERVE(NCT00565409)	2008.3.6-2009.9.9	3	52	46.4	6·4	157	202	34	etanercept 25mg qw+MTX
					48.1	6·8	164	202		etanercept 50mg qw+MTX
					48.3	7·3	167	200		placebo+MTX
Keystone et al. (67)	CAMEO (NCT00654368)	2008.6-2012.12	6	104	54.3	9.0	72	98	205	etanercept 50 mg qw
					54.4	9.3	84	107		etanercept 50 mg qw + MTX
van Vollenhovn et al. (70)	NCT00858780	NR	4	20	53.8	11.5	17	23	73	etanercept50mg qw + MTX
					59.6	16.6	18	27		etanercept25mg qw + MTX
					56.1	12.3	16	23		placebo +MTX
Yamanaka et al. (71)	ENCOURAGE (UMIN000002687)	2009.8-2014.4	5	52	52.8	2.0	138	161	191	etanercept 25 mg biw + MTX
					54.6	1.9	25	30		MTX
Pavelka et al. (73)	NCT01578850	2012.7-2015.3	6	28	46.1	8.0	136	167	343	etanercept 50mg qw +DMARDs
					47.2	8.3	143	176		placebo +DMARDs
Curtis et al. (82)	SEAM- RA	2015.2.20-2018.6.26	6	48	56.2	9.7	76	101	153	MTX
					54.8	11.0	77	101		etanercept
					55.9	10.3	40	51		MTX + etanercept
Kay et al. (30)		2003.12.1-2006.2.21	5	20	52	5.6	26	35	172	placebo + MTX
					57	8.2	30	35		50mg golimumab q4w + MTX
					48	8.2	23	34		50mg golimumab eow + MTX
					57.5	6.3	26	34		100mg golimumab eow + MTX
					53.5	9.0	27	34		50mg golimumab eow + MTX
Emery et al. (34)	GO-BEFORE	2005.12.12-2007.10.1	6	24	50.9	3.5	135	159	634	Golimumab 50 mg q4w + MTX
					50.2	3.6	125	159		Golimumab 100 mg q4w + MTX
					48.2	4.1	159	159		Golimumab 100 mg q4w + Placebo
					48.6	2.9	134	160		Placebo+MTX
Keystone et al. (36)	GO-FORWARD	20005.12.19-2007.9.17	5	16	52	4.5	72	89	444	Golimumab 50 mg q4w + MTX
					50	6.7	72	89		Golimumab 100 mg q4w + MTX
					51	5.9	105	133		Golimumab 100 mg q4w + Placebo
					52	6.5	109	133		Placebo+MTX
Smolen et al. (38)	GO-AFTER (NCT00299546)	2006.2.21-2007.9.26	7	16	55	9.6	113	153	461	Golimumab 50 mg q4w
					55	8.7	122	153		Golimumab 100 mg q4w
					54	9.8	132	155		Placebo
Kremer et al. (40)	NCT00361335	2006.8.24-2008.8.25	6	16	49.9	7.4	21	128	643	Golimumab 2mg/kg q12w
					48.4	8.4	10	129		Golimumab 4mg/kg q12w
					49.7	8.1	30	129		Golimumab 2mg/kg q12w + MTX
					49.6	9.4	25	128		Golimumab 4mg/kg q12w + MTX
					50.2	7.4	24	129		Placebo + MTX
Tanaka et al. (45)	GO-FORTH	2008.5-2009.11	5	16	50.4	8.8	15	86	261	Golimumab 50 mg q4w + MTX
					50	8.1	78	87		Golimumab 100 mg q4w + MTX
					51.1	8.7	73	88		Placebo + MTX
Takeuchi et al. (53)	GO-MONO	NA	4	16	52.9	8.1	81	101	308	Golimumab 50 mg q4w
					51.6	9.4	85	102		Golimumab 100 mg q4w
					52.4	9.2	86	105		Placebo
Weinblatt et al. (55)	GO-FURTHER(NCT00973479)	2009.9.14-2011.5.18	7	16	51.4	7.0	157	197	592	Placebo +MTX
					51.9	6.9	326	395		Golimumab2 mg/kg+MTX
Li et al. (68)	NCT01248780	2010.8-2012.7	4	24	47.7	7.6	110	132	264	Golimumab 50 mg q4w + MTX
					46.7	8.0	104	132		Placebo + MTX
Maini et al. (17)		1997.3.31-2000.3.9	7	102	54	10	70	86	428	infliximab 3mg/kg, q8w+MTX
					52	9	66	86		infliximab 3mg/kg, q4w+MTX
					54	11	67	87		infliximab 10mg/kg, q8w+MTX
					52	12	59	81		infliximab 10mg/kg, q4w+MTX
					51	11	70	88		placebo +MTX
St. Clair et al. (18)		2000.7.21-2002.2.28	7	54	51	0.8	255	359	1004	infliximab 3mg/kg, q8w+MTX
					50	0.9	247	363		infliximab 6mg/kg, q8w+MTX
					50	0.9	212	282		placebo +MTX
Abe et al. (12)		2000.4.19-2000.10.27	4	6	55.2	9.1	40	49	147	infliximab 3mg/kg, q8w+MTX
					56.8	7.1	40	51		infliximab 10mg/kg, q8w+MTX
					55.1	7.5	35	47		placebo +MTX
Westhoven et al. (23)	START	2001.9-2003.11	6	22	53	7.8	288	360	1082	infliximab 3mg/kg +MTX
					52	6.3	281	361		infliximab 10mg/kg +MTX
					52.0	8.4	302	361		placebo+MTX
Zhang et al. (24)		2003.7-2004.7	4	18	47.9	NR	13	87	173	infliximab (Remicade, Centocor) at a dose of 3 mg/kg body weight qw 0, 2, 6 and 14.
					48.9	NR	13	86		placebo
Schiff et al. (32)	ATTEST	2005.2-2007.2	6	28	49.1	7.3	136	165	275	infliximab 3mg/kg, q8w+MTX
					49.4	8.4	96	110		placebo +MTX
Kim et al. (50)	NCT00202852, NCT00732875	2005.6-2006.5	5	30	49.3	7.4	64	69	138	Infliximab
					51.4	9.8	64	69		placebo
Leirisalo-Repo et al. (51)	NCT00908089	2003.3-2005.4	6	102	47	0.33	35	50	3403	infliximab
					46	0.33	31	49		placebo

biw, twice a week; qw, weekly; eow, every two weeks; q4w, every four weeks; q8w, every 8 weeks; q12w, every 12 weeks; MTX, methotrexate; DMARD, disease-modifying anti-rheumatic drugs; NA, not re.

### Adverse Events

**Figure 2:**
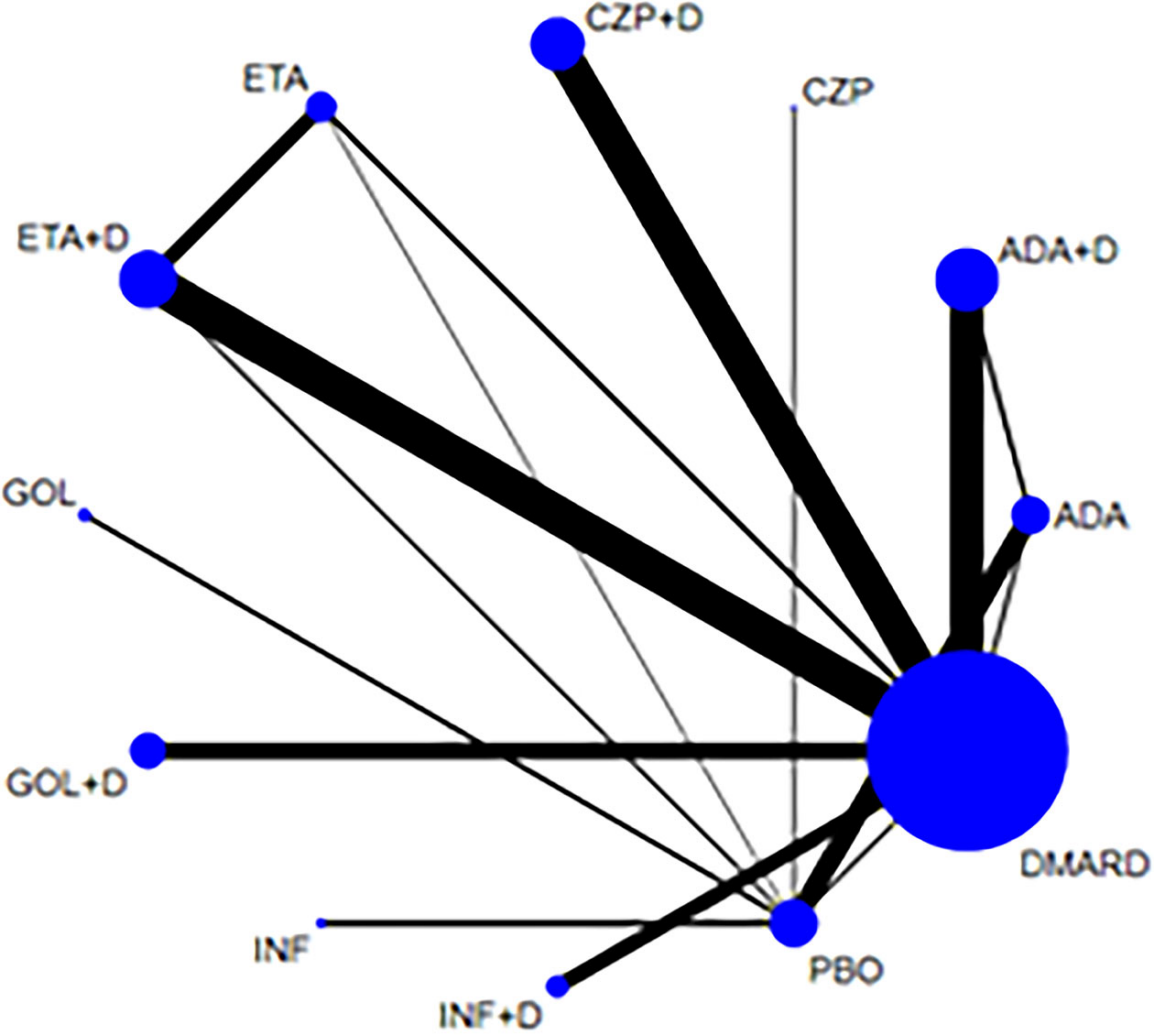
Network of treatment comparisons for adverse events. The size of the circles corresponds to the total number of people. Direct comparable treatments are connected with a line. ADA, adalimumab; + D, plus DMARD; CZP, certolizumab; ETA, etanercept; GOL, golimumab; INF, infliximab; PBO, placebo; DMARD, disease-modifying anti-rheumatic drugs.

**Figure 3:**
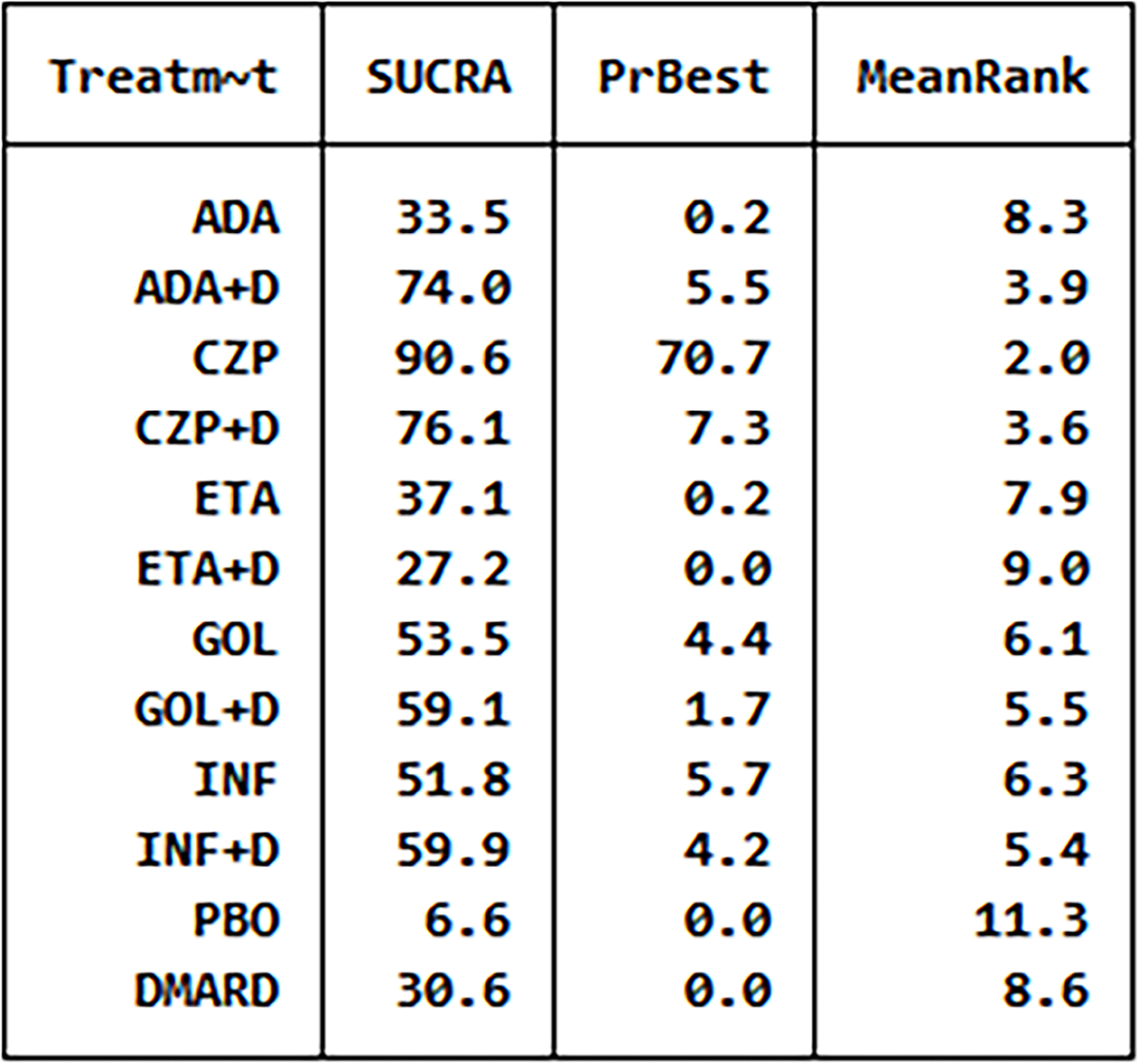
The analysis SUCRA of adverse events for 12 therapies. ADA, adalimumab; + D, plus DMARD; CZP, certolizumab; ETA, etanercept; GOL, golimumab; INF, infliximab; PBO, placebo; DMARD, disease-modifying anti-rheumatic drugs.

58 articles (12, 15, 16, 19, 21–26, 28–38, 40–42, 44–47, 49–56, 58–69, 71–75, 77, 79–82) reported the occurrence of AEs and 23778 RA patients was included. The network of eligible comparisons is shown in **Figure 2**. Network meta-analysis showed that adalimumab combined DMARDs compared with placebo therapy statistically significantly increased the risk of AEs by 60% (1.60, 95% CI: 1.06, 2.42); compared with DMARDs, the risk of AEs increased by 28% (1.28, 95% CI: 1.08, 1.52) (**Table 2** and **Figure 3**). Certolizumab also found that compared with placebo therapy, the risk of AE increased by 127% (2.27, 95% CI: 1.22, 4.24). In addition, certolizumab combined DMARDs compared with placebo therapy statistically significantly increased the risk of AEs by 63% (1.63, 95% CI: 1.07, 2.46); compared with DMARDs, the risk of AEs increased by 30% (1.30, 95% CI: 1.10, 1.54). Comparisons between treatments showed certolizumab combined DMARDs increased the risk of AEs compared with etanercept combined DMARDs (1.34, 95% CI: 1.05, 1.70); adalimumab combined DMARDs increased the risk of AEs compared with etanercept combined DMARDs (1.32, 95% CI: 1.03, 1.67) (**Table 2**). There was no statistically significant difference between other comparisons.

**Table 2:** OR of adverse events for 12 therapies.

**ADA**	1.31 (0.83,2.07)	1.86 (0.93,3.71)	1.33 (0.84,2.12)	1.04 (0.68,1.60)	1.00 (0.64,1.55)	1.16 (0.72,1.85)	1.20 (0.74,1.92)	1.15 (0.65,2.03)	1.21 (0.71,2.06)	0.82 (0.61,1.10)	1.02 (0.66,1.58)
0.76 (0.48,1.20)	**ADA+DMARD**	1.42 (0.67,3.00)	1.02 (0.80,1.29)	0.79 (0.57,1.10)	0.76 (0.60,0.97)	0.88 (0.50,1.55)	0.91 (0.70,1.18)	0.88 (0.46,1.67)	0.92 (0.65,1.30)	0.62 (0.41,0.94)	0.78 (0.66,0.92)
0.54 (0.27,1.07)	0.70 (0.33,1.49)	**CZP**	0.72 (0.34,1.51)	0.56 (0.27,1.15)	0.54 (0.26,1.11)	0.62 (0.30,1.31)	0.64 (0.30,1.37)	0.62 (0.28,1.37)	0.65 (0.30,1.42)	0.44 (0.24,0.82)	0.55 (0.26,1.14)
0.75 (0.47,1.20)	0.98 (0.78,1.25)	1.40 (0.66,2.96)	**CZP+DMARD**	0.78 (0.56,1.09)	0.75 (0.59,0.95)	0.87 (0.49,1.54)	0.90 (0.69,1.17)	0.86 (0.45,1.64)	0.91 (0.65,1.27)	0.62 (0.41,0.93)	0.77 (0.65,0.91)
0.96 (0.63,1.48)	1.26 (0.91,1.75)	1.79 (0.87,3.69)	1.28 (0.92,1.78)	**ETA**	0.96 (0.73,1.25)	1.11 (0.65,1.90)	1.15 (0.81,1.63)	1.11 (0.60,2.04)	1.16 (0.77,1.75)	0.79 (0.55,1.14)	0.98 (0.74,1.30)
1.00 (0.65,1.56)	1.32 (1.03,1.67)	1.87 (0.90,3.88)	1.34 (1.05,1.70)	1.04 (0.80,1.36)	**ETA+DMARD**	1.16 (0.67,2.01)	1.20 (0.92,1.56)	1.15 (0.62,2.14)	1.21 (0.86,1.70)	0.82 (0.56,1.20)	1.02 (0.86,1.21)
0.86 (0.54,1.38)	1.13 (0.64,1.99)	1.61 (0.77,3.38)	1.15 (0.65,2.04)	0.90 (0.53,1.53)	0.86 (0.50,1.49)	**GOL**	1.03 (0.58,1.84)	0.99 (0.53,1.87)	1.04 (0.56,1.95)	0.71 (0.47,1.06)	0.88 (0.51,1.52)
0.84 (0.52,1.34)	1.10 (0.85,1.42)	1.56 (0.73,3.32)	1.11 (0.86,1.44)	0.87 (0.61,1.23)	0.83 (0.64,1.09)	0.97 (0.54,1.73)	**GOL+DMARD**	0.96 (0.50,1.85)	1.01 (0.70,1.45)	0.69 (0.45,1.05)	0.85 (0.70,1.04)
0.87 (0.49,1.54)	1.14 (0.60,2.17)	1.62 (0.73,3.58)	1.16 (0.61,2.20)	0.90 (0.49,1.67)	0.87 (0.47,1.61)	1.01 (0.53,1.90)	1.04 (0.54,2.00)	**INF**	1.05 (0.53,2.09)	0.71 (0.44,1.16)	0.89 (0.48,1.65)
0.83 (0.49,1.41)	1.09 (0.77,1.53)	1.54 (0.70,3.39)	1.10 (0.79,1.55)	0.86 (0.57,1.30)	0.83 (0.59,1.16)	0.96 (0.51,1.79)	0.99 (0.69,1.42)	0.95 (0.48,1.89)	**INF+DMARD**	0.68 (0.42,1.10)	0.85 (0.63,1.14)
1.22 (0.91,1.63)	1.60 (1.06,2.42)	2.27 (1.22,4.24)	1.63 (1.07,2.46)	1.27 (0.88,1.83)	1.22 (0.83,1.78)	1.41 (0.95,2.11)	1.46 (0.95,2.25)	1.40 (0.86,2.29)	1.47 (0.91,2.38)	**PBO**	1.25 (0.85,1.82)
0.98 (0.63,1.51)	1.28 (1.08,1.52)	1.82 (0.88,3.79)	1.30 (1.10,1.54)	1.02 (0.77,1.35)	0.98 (0.82,1.16)	1.13 (0.66,1.96)	1.17 (0.96,1.43)	1.13 (0.60,2.10)	1.18 (0.88,1.59)	0.80 (0.55,1.17)	**DMARD**

Results below the diagonal are the rate ratios with 95% confidence intervals from the network meta-analysis of direct and indirect comparisons between the row-defining treatment and the column-defining treatment. Numbers in red highlight statistically significant results. ADA, adalimumab; + D, plus DMARD; CZP, certolizumab; ETA, etanercept; GOL, golimumab; INF, infliximab; PBO, placebo; DMARD, disease-modifying anti-rheumatic drugs.

**Figure 4:**
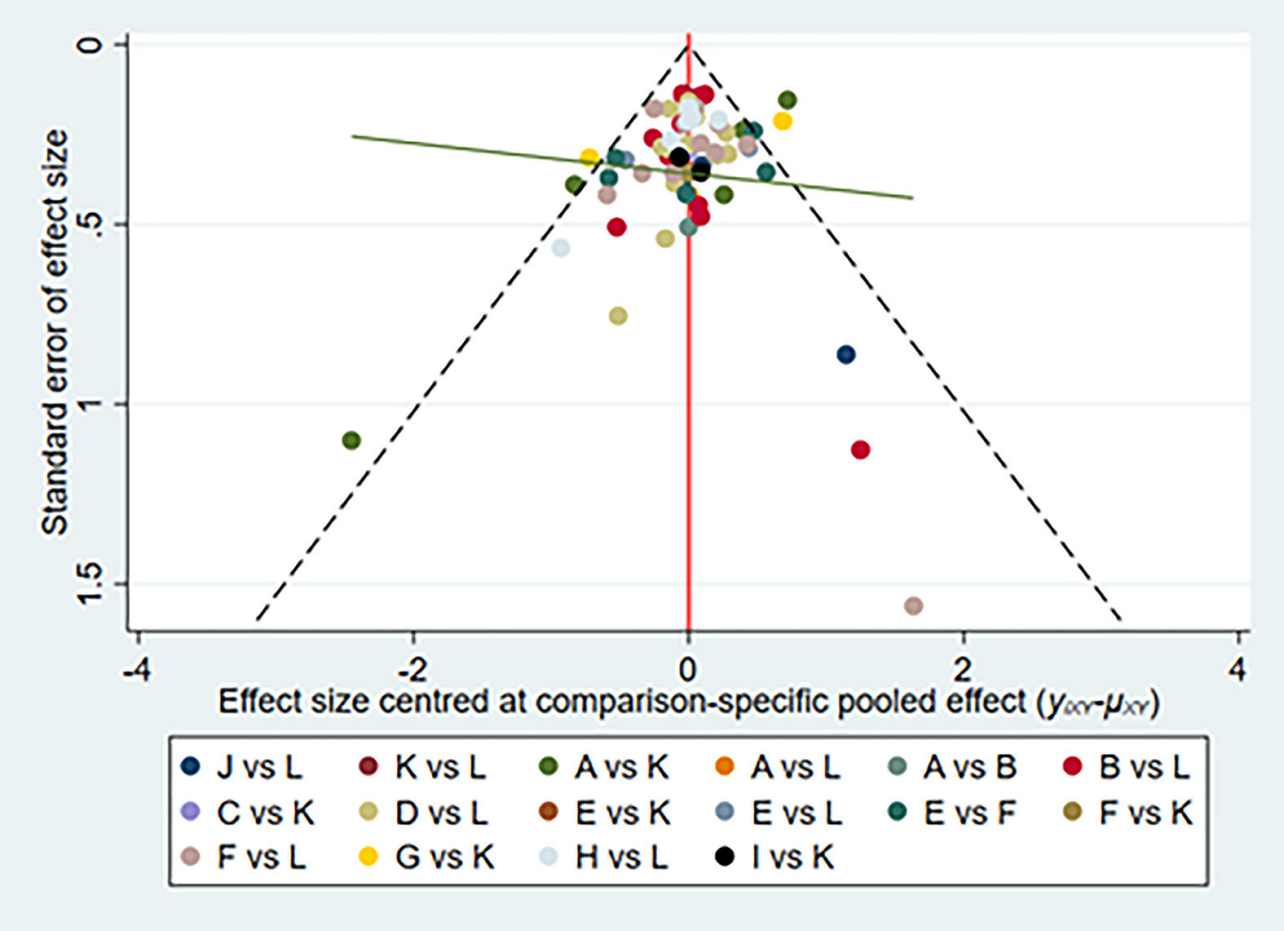
Network of funnel plot for adverse events. A, adalimumab; B, adalimumab + DMARD; C, certolizumab; D, certolizumab + DMARD; F, etanercept; G, etanercept + DMARD; H, golimumab; I, golimumab + DMARD; J, infliximab; K, infliximab + DMARD; L, DMARD; DMARD, disease-modifying anti-rheumatic drugs.

We have made global consistency. The test result p-value was 0.9095, so the consistency model could be used. We also established local consistency and the p-value of the test result exceeded 0.05, which was considered local. We analyzed SUCRA to research the probability of adverse events for each therapy. The results indicated that certolizumab had the highest probability to cause AEs (SUCRA = 0.906), while PBO had the lowest probability to cause AEs (SUCRA = 0.066) compared with the other therapies (**Figure 3**). There was a funnel plot with no obvious asymmetry, indicating no publication bias (**Figure 4**).

### Serious Adverse Events

**Figure 5:**
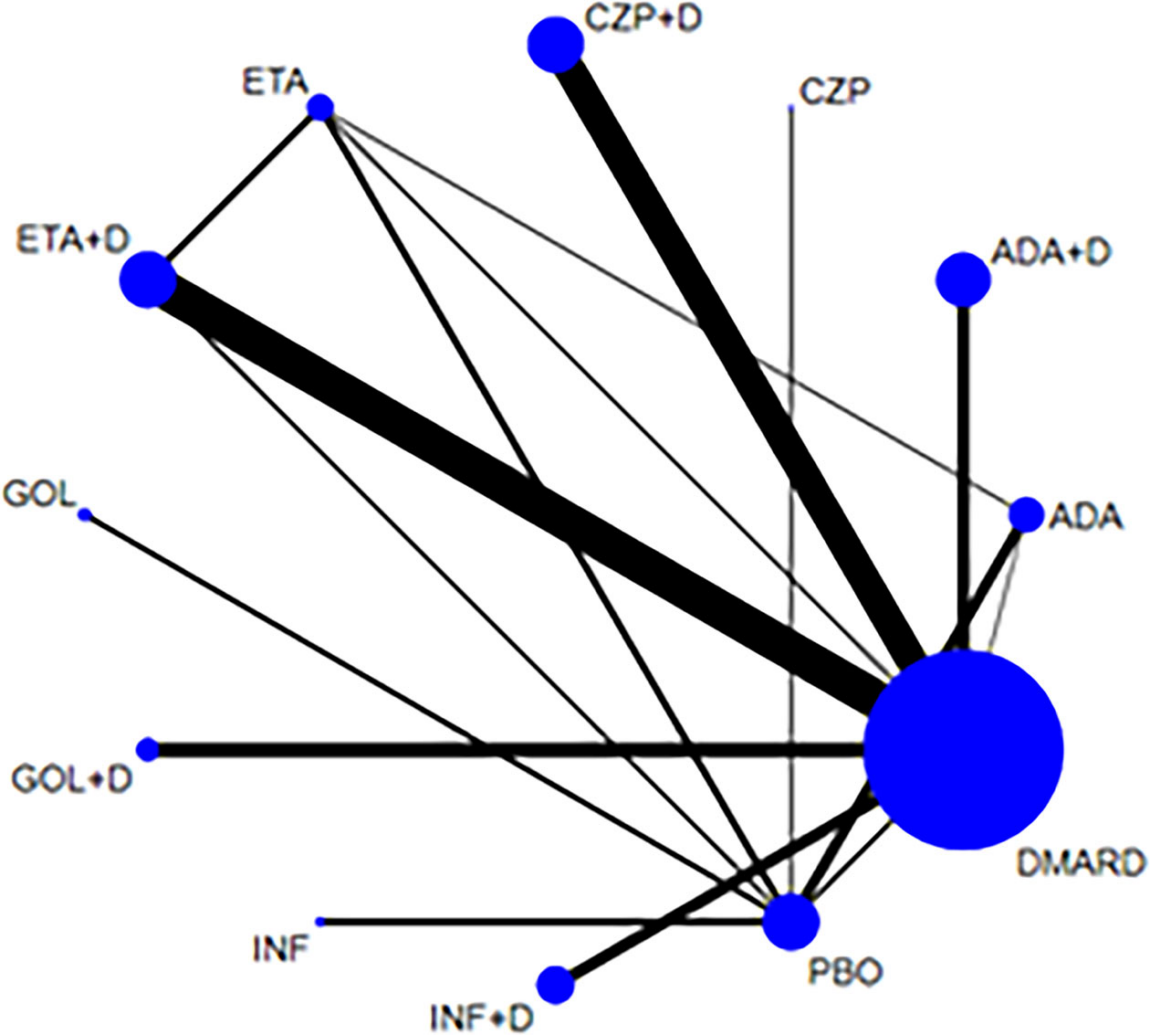
Network of treatment comparisons for serious adverse events. The size of the circles corresponds to the total number of people. Direct comparable treatments are connected with a line. ADA, adalimumab; + D, plus DMARD; CZP, certolizumab; ETA, etanercept; GOL, golimumab; INF, infliximab; PBO, placebo; DMARD, disease-modifying anti-rheumatic drugs.

58 articles (12, 13, 15, 17–19, 22, 24–27, 29–32, 34–36, 38, 40–52, 54, 56–60, 62–70, 72–82) reported the occurrence of SAEs and 23805 RA patients was included. The network of eligible comparisons was shown in **Figure 5**. Network meta-analysis showed that golimumab combined DMARDs compared with placebo therapy statistically significantly increased the risk of SAEs by 227% (3.27, 95% CI: 1.08, 9.92); Compared with DMARDs, the risk of SAEs increased by 170% (2.70, 95% CI: 1.15, 6.32). Comparisons between treatments showed adalimumab (0.20, 95% CI: 0.07, 0.59), etanercept(0.35, 95% CI: 0.12, 1.00), etanercept combined DMARDs (0.39, 95% CI: 0.15, 0.96), golimumab (0.19, 95% CI: 0.05, 0.77) decreased the risk of SAEs compared with golimumab combined DMARDs; adalimumab (0.39, 95% CI: 0.18, 0.84) decreased the risk of SAEs compared with certolizumab combined DMARDs; golimumab combined DMARDs increased the risk of SAEs compared with infliximab (6.50, 95% CI: 1.41, 29.90) (**Table 3**). There was no statistically significant difference between other comparisons.

**Table 3:** OR of serious adverse events of 12 therapies.

**ADA**	2.05 (0.94,4.49)	4.27 (0.94,19.46)	2.57 (1.19,5.56)	1.80 (0.94,3.42)	1.96 (0.97,3.97)	0.96 (0.36,2.60)	5.08 (1.68,15.30)	0.78 (0.24,2.49)	2.20 (1.00,4.81)	1.55 (0.94,2.56)	1.88 (0.93,3.80)
0.49 (0.22,1.07)	**ADA+DMARD**	2.08 (0.40,10.71)	1.25 (0.78,2.02)	0.88 (0.44,1.75)	0.95 (0.59,1.54)	0.47 (0.15,1.52)	2.48 (0.99,6.22)	0.38 (0.10,1.42)	1.07 (0.65,1.75)	0.76 (0.34,1.68)	0.92 (0.65,1.30)
0.23 (0.05,1.07)	0.48 (0.09,2.47)	**CZP**	0.60 (0.12,3.08)	0.42 (0.09,1.99)	0.46 (0.09,2.27)	0.23 (0.04,1.20)	1.19 (0.19,7.30)	0.18 (0.03,1.08)	0.51 (0.10,2.65)	0.36 (0.09,1.53)	0.44 (0.09,2.19)
0.39 (0.18,0.84)	0.80 (0.49,1.29)	1.66 (0.32,8.50)	**CZP+DMARD**	0.70 (0.35,1.38)	0.76 (0.48,1.21)	0.38 (0.12,1.20)	1.98 (0.79,4.92)	0.30 (0.08,1.12)	0.85 (0.53,1.38)	0.61 (0.28,1.32)	0.73 (0.53,1.02)
0.56 (0.29,1.06)	1.14 (0.57,2.29)	2.38 (0.50,11.25)	1.43 (0.72,2.83)	**ETA**	1.09 (0.62,1.91)	0.54 (0.19,1.53)	2.83 (1.00,8.02)	0.43 (0.13,1.45)	1.22 (0.61,2.45)	0.87 (0.47,1.58)	1.05 (0.57,1.91)
0.51 (0.25,1.04)	1.05 (0.65,1.69)	2.18 (0.44,10.77)	1.31 (0.83,2.08)	0.92 (0.52,1.61)	**ETA+DMARD**	0.49 (0.16,1.50)	2.59 (1.04,6.47)	0.40 (0.11,1.41)	1.12 (0.69,1.82)	0.79 (0.39,1.61)	0.96 (0.69,1.34)
1.04 (0.38,2.80)	2.13 (0.66,6.85)	4.42 (0.83,23.50)	2.66 (0.83,8.50)	1.86 (0.65,5.32)	2.03 (0.67,6.15)	**GOF**	5.26 (1.29,21.45)	0.81 (0.21,3.13)	2.28 (0.70,7.36)	1.61 (0.68,3.80)	1.95 (0.64,5.97)
0.20 (0.07,0.59)	0.40 (0.16,1.01)	0.84 (0.14,5.15)	0.51 (0.20,1.26)	0.35 (0.12,1.00)	0.39 (0.15,0.96)	0.19 (0.05,0.77)	**GOF+DMARD**	0.15 (0.03,0.71)	0.43 (0.17,1.08)	0.31 (0.10,0.93)	0.37 (0.16,0.87)
1.28 (0.40,4.08)	2.63 (0.71,9.76)	5.46 (0.93,32.24)	3.29 (0.89,12.15)	2.30 (0.69,7.70)	2.51 (0.71,8.85)	1.24 (0.32,4.79)	6.50 (1.41,29.90)	**INF**	2.81 (0.76,10.45)	1.99 (0.70,5.67)	2.41 (0.68,8.55)
0.46 (0.21,1.00)	0.93 (0.57,1.53)	1.94 (0.38,10.02)	1.17 (0.72,1.89)	0.82 (0.41,1.64)	0.89 (0.55,1.45)	0.44 (0.14,1.42)	2.31 (0.92,5.79)	0.36 (0.10,1.32)	**INF+DMARD**	0.71 (0.32,1.57)	0.86 (0.61,1.21)
0.64 (0.39,1.06)	1.32 (0.60,2.92)	2.74 (0.65,11.51)	1.65 (0.76,3.61)	1.16 (0.63,2.11)	1.26 (0.62,2.55)	0.62 (0.26,1.46)	3.27 (1.08,9.92)	0.50 (0.18,1.43)	1.41 (0.64,3.13)	**PBO**	1.21 (0.59,2.47)
0.53 (0.26,1.07)	1.09 (0.77,1.55)	2.27 (0.46,11.25)	1.37 (0.98,1.90)	0.95 (0.52,1.74)	1.04 (0.75,1.45)	0.51 (0.17,1.57)	2.70 (1.15,6.32)	0.41 (0.12,1.47)	1.17 (0.83,1.65)	0.83 (0.40,1.69)	**DMARD**

Results below the diagonal are the rate ratios with 95% confidence intervals from the network meta-analysis of direct and indirect comparisons between the row-defining treatment and the column-defining treatment. Numbers in red highlight statistically significant results. ADA, adalimumab; + D, plus DMARD; CZP, certolizumab; ETA, etanercept; GOL, golimumab; INF, infliximab; PBO, placebo; DMARD, disease-modifying anti-rheumatic drug.

**Figure 6:**
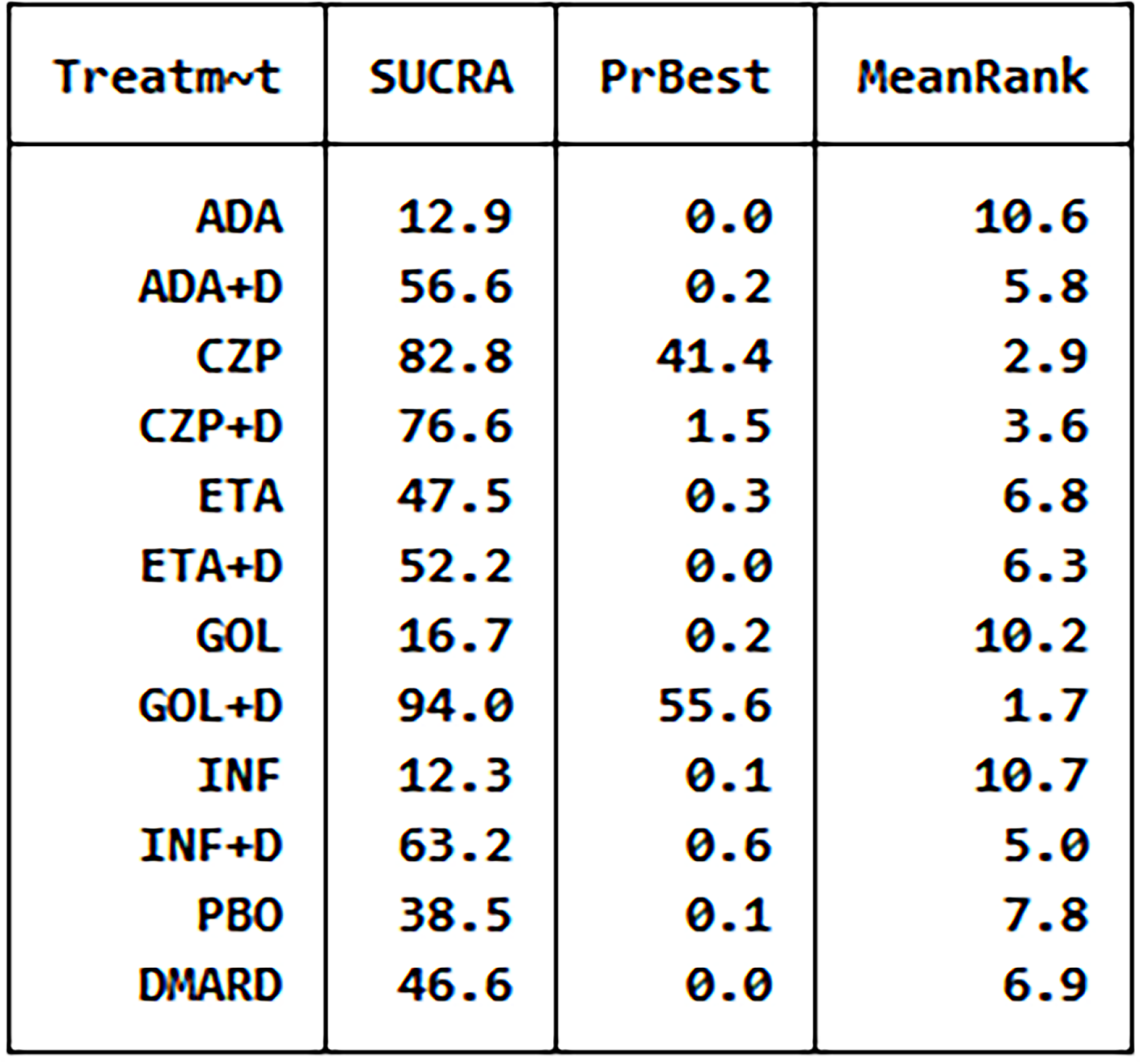
The analysis SUCRA of serious adverse events for 12 therapies. ADA, adalimumab; + D, plus DMARD; CZP, certolizumab; ETA, etanercept; GOL, golimumab; INF, infliximab; PBO, placebo; DMARD, disease-modifying anti-rheumatic drugs.

**Figure 7:**
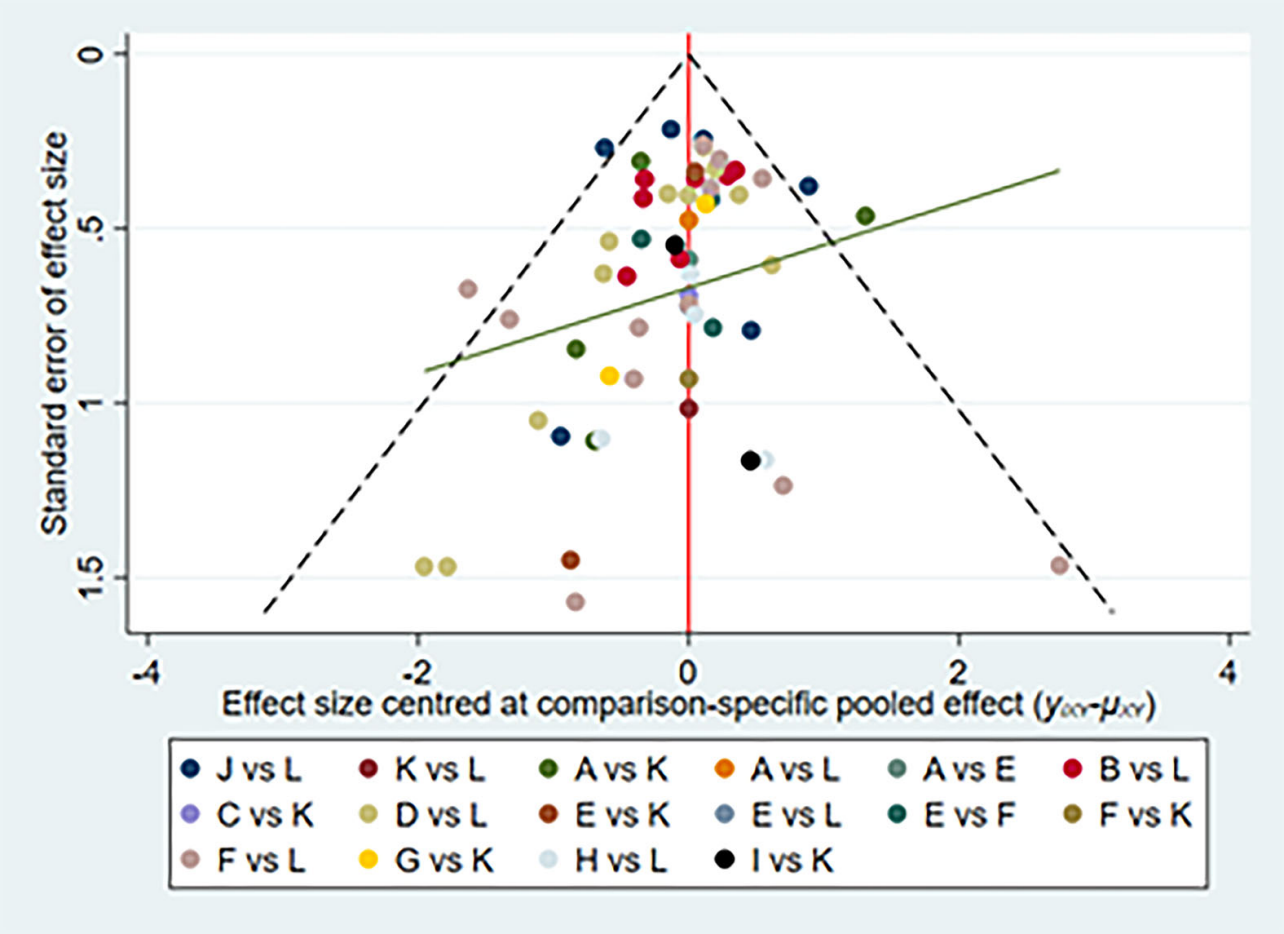
Network of funnel plot for serious adverse events. A, adalimumab; B, adalimumab + DMARD; C, certolizumab; D, certolizumab + DMARD; F, etanercept; G, etanercept + DMARD; H, golimumab; I, golimumab + DMARD; J, infliximab; K, infliximab + DMARD; L, DMARD; DMARD, disease-modifying anti-rheumatic drugs.

We did the global consistency test. The test result p-value was 0.8840. We also made local consistency and the test result p-value was greater than 0.05, which was considered to be locally consistent. According to the SUCRA analysis, golimumab combined DMARDs had the highest risk to cause SAEs (SUCRA = 0.940), while adalimumab had the lowest risk to cause SAEs (SUCRA = 0.130) compared with the other 11 therapies (**Figure 6**). There was a funnel plot asymmetry, with the right corner of the pyramidal part of the funnel missing, which suggested a possible bias (**Figure 7**).

### Infections

40 articles (12, 15, 17, 22, 25–28, 30, 31, 33, 34, 36, 38, 40–42, 45, 49, 54–56, 58–60, 62–66, 72–77, 79–82) reported the occurrence of AEs and 15285 RA patients was included. The network of eligible comparisons was shown in the **Supplementary Figure 1**. Network meta-analysis showed that golimumab combined DMARDs compared with DMARDs increased the risk of infections by 35% (1.35, 95% CI: 1.10, 1.66); infliximab combined DMARDs compared with DMARDs increased the risk of infections by 102% (2.02, 95% CI: 1.31, 3.11). Comparisons between treatments showed adalimumab combined DMARDs (0.59, 95% CI: 0.37, 0.95), etanercept(0.49, 95% CI: 0.28, 0.88), etanercept combined DMARDs (0.56, 95% CI: 0.35, 0.91), golimumab combined DMARDs (0.51, 95% CI: 0.31, 0.83) decreased the risk of infections compared with infliximab combined DMARDs (**supplementary Table 1**). There was no statistically significant difference between other comparisons.

We did the global consistency test. The test result p-value was 0.6713. We also established local consistency and the p-value of the test result exceeded 0.05, which was considered local. According to the SUCRA analysis, infliximab combined DMARDs had the highest risk to cause infections (SUCRA = 0.910), while DMARDs had the lowest risk to cause infections SUCRA = 0.210) compared with the other 11 therapies (**Supplementary Figure 2**). There was a funnel plot (**Supplementary Figure 3**) with no obvious asymmetry, indicating no publication bias.

### Serious Infections

55 articles (11–20, 22, 23, 26–38, 40, 42, 45, 47–49, 51, 52, 54, 56–60, 62–66, 68, 69, 72–77, 80–82) reported the occurrence of serious infections, involving a total of 24740 RA patients. The network of eligible comparisons was shown in the **Supplementary Figure 4**. Network meta-analysis showed that there was no statistically significant difference between 12 therapies (**Supplementary Table 2**).

We did the global consistency test. The resulting p-value was 0.4900. We also made local consistency and the test result p-value was greater than 0.05, which was considered to be locally consistent. According to the SUCRA analysis, certolizumab had the highest risk to cause serious infections (SUCRA =0.817), while etanercept combined DMARDs had the lowest risk to cause serious infections (SUCRA = 0.285) compared with the other 11 therapies (**Supplementary Figure 5**). There was a funnel plot asymmetry, with the right corner of the pyramidal part of the funnel missing, which suggested a possible bias (**Supplementary Figure 6**).

### Malignant Tumors

32 articles (14–20, 23, 26, 27, 29–32, 34–39, 43, 47–49, 52, 57, 60, 65, 74, 75, 77, 79) reported the occurrence of malignant tumors, involving 16947 RA patients. The network of eligible comparisons was shown in the **Supplementary Figure 7**. Mesh meta-analysis showed that there was no statistically significant difference between 12 therapies (**Supplementary Table 3**).

We did the global consistency test. The test result p-value was 0.6219. We also made local consistency and the test result p-value was greater than 0.05, which was considered to be locally consistent. According to the SUCRA analysis (**Supplementary Figure 8**), golimumab had the highest risk to cause malignant tumors (SUCRA =0.778), while golimumab combined DMARDs had the lowest risk to cause malignant tumors (SUCRA = 0.285) compared with the other 11 therapies.

## Discussion

Based on the data and information of included RCTs, our study aims to evaluate the risk of adverse effects of 10 anti-TNF therapies in patients with rheumatoid arthritis. All available direct and indirect evidence of various treatment options was analyzed and compared simultaneously by network meta-analysis, which has a great advantage over traditional meta-analysis and makes up for the lack of head-to-head comparisons (83). To comprehensively assess the safety of anti-TNF therapies in RA patients, we also pay attention to detailed AEs like infections, serious infections, malignant tumors. What’s more, our meta-analysis included all RCTs with medium or high quality more recent studies to August 2021, which avoided the deficiency of observational studies and low-quality studies. Therefore, our studies are much more reliable than the other meta-analyses or network meta-analyses.

After analysis of 10 therapies for patients with RA from 72 RCTs, we found golimumab monotherapy, infliximab monotherapy, etanercept monotherapy, adalimumab monotherapy, and etanercept+DMARDs therapy are the safer treatments when the efficacies are similar, they did not increase the risk of all analyzed safety indexes. A comprehensive analysis of the results of network meta-analysis and SUCRA sequencing diagram of adverse reactions showed that etanercept monotherapy is the safest therapy of the 10 therapies was etanercept monotherapy. Etanercept monotherapy was recommended as an alternative treatment due to its good safety outcomes. Certolizumab+DMARDs was considered the worst therapy, so it was necessary to avoid using this therapy. Besides, etanercept may be able to reduce the expression and production of vascular endothelial growth factor, NO, and inducible NO synthase and contribute to having a beneficial effect upon the progression of atherosclerosis, reducing the risk of acute cardiovascular and/or cerebrovascular events (84). This is further demonstrated that etanercept therapy is safer. In 2014, Murdaca et al. investigated the role of single-nucleotide polymorphisms (SNPs) at positions -238, - 308, and + 489 of the TNF-a gene in the response to TNF-a inhibitors (adalimumab, etanercept, or infliximab) and found that the SNP + 489 G allele may promote the response to etanercept. Thus, genetic polymorphisms could be performed before treatment to determine suitability for the etanercept monotherapy (85).

After head-to-head comparisons for the effects of these 10 anti-TNF therapies on the risk of serious infections, malignant tumors, we found no difference of 10 therapies. And compared with PBO therapy or DMARDs therapy, these 10 anti-TNF therapies did not affect the risk of serious infections, malignant tumors, and tuberculosis infection. This may be indicated that these 10 anti-TNF therapies are safe for serious infections, malignant tumors, and tuberculosis infection.

Interestingly, among these 10 anti-TNF therapies, five are TNF-a inhibitor monotherapies and another five are TNF-α inhibitors combinations of DMARDs. It was easy to find that in most cases the safety of TNF-α inhibitor monotherapy was superior to the corresponding TNF-α inhibitors combinations of DMARDs. For example, the SUCRAs of safety outcomes for golimumab+ DMARDs are as follows: 59.1% (AEs), 94.0% (SAEs), and 57.5% (serious infections). By contrast, golimumab monotherapy was safer with corresponding SUCRAs of 53.5%, 16.7%, and 31.8%. Previous researchers have also conducted comparisons between TNF-α inhibitor monotherapy and TNF-α inhibitor combined with MTX. For instance, Breedveld et al. demonstrated that the proportions of RA patients inducing AEs and serious infections were higher under the treatment of adalimumab + DMARDs than the adalimumab monotherapy, which was in line with our results. However, some studies published before also presented no difference between the two kinds of treatment groups (86). Patients with RA treated with etanercept and those treated with etanercept + DMARDs were similar. Thus, further research should be conducted to estimate whether TNF-α inhibitor combined with DMARDs therapy benefits TNF-α inhibitor monotherapy or not.

Although we have made the study as comprehensive as possible, there are still some limitations. Firstly, even though the included trials were all RCTs, the results of safety comparisons among 10 drug therapies still showed some statistical inconsistency. Perhaps the RCTs with contradictions between direct and indirect evidence should be reconsidered. Secondly, 22 trials only had a follow-up time of fewer than 20 weeks. A short duration was not enough to judge the safety of treatment. Thirdly, medication dose, treatment cost, patient compliance, and other influential factors also affected trial homogeneity. Last but not least, different RCTs included in our research had different definitions of safety outcomes. There was still a shortage of clear definitions of AEs and SAEs.

In conclusion, we regard etanercept monotherapy as the optimal choice for RA patients in clinical practice when the efficacy was similar. Conversely, certolizumab+DMARDs therapy was not recommended. It was necessary to conduct long-term studies on patients with RA to provide a more complete assessment of diverse treatments and make a more judicious choice in clinical practice. All efforts should be made to improve the life quality and health standards for patients with RA.

## Data Availability Statement

The original contributions presented in the study are included in the article/**Supplementary Material**. Further inquiries can be directed to the corresponding author.

## Author Contributions

W-xP, YL, and BH conceived this meta-analysis. YL and XC extracted data. H-rX provided statistical advice and Q-zZ did all statistical analyses. YL, BH, H-rX, and XC checked for statistical inconsistency and interpreted data. YL, BH, and W-wL contributed to data interpretation. YL, BH, and JH drafted the report. H-rX, XC, and JH critically reviewed the article. All authors read and approved the final manuscript.

## Conflict of Interest

The authors declare that the research was conducted in the absence of any commercial or financial relationships that could be construed as a potential conflict of interest.

## Publisher’s Note

All claims expressed in this article are solely those of the authors and do not necessarily represent those of their affiliated organizations, or those of the publisher, the editors and the reviewers. Any product that may be evaluated in this article, or claim that may be made by its manufacturer, is not guaranteed or endorsed by the publisher.
