# Supplementary material for: The Risk of Adverse Effects of TNF-α Inhibitors in Patients With Rheumatoid Arthritis: A Network Meta-Analysis
Source: Front Immunol. 2022 Feb 16;13:814429. doi: 10.3389/fimmu.2022.814429 (PMC8888889; doi:10.3389/fimmu.2022.814429)
Supplement: Supplementary file 1 [file DataSheet_1.docx]

Supplementary Material

# Supplementary Figures and Tables

## Supplementary Figures


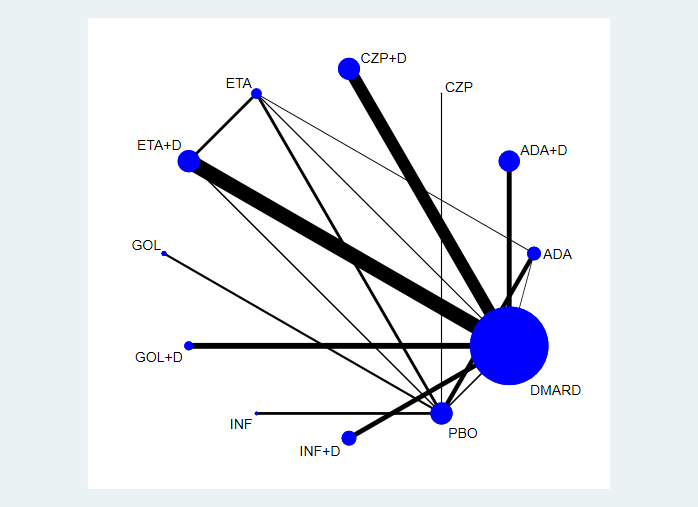


**Supplementary Figure 1.** Network of treatment comparisons for infections. The size of the circles corresponds to the total number of people. Direct comparable treatments are connected with a line. ADA: adalimumab; + D: plus DMARD. CZP: certolizumab; ETA: etanercept; GOL: golimumab; INF: infliximab; PBO: placebo; DMARD: disease-modifying anti-rheumatic drugs.


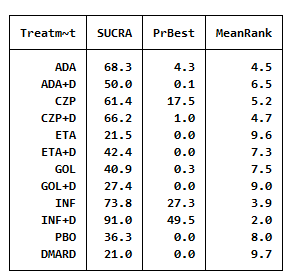


**Supplementary Figure 2** The analysis SUCRA of infections for 12 therapies. ADA: adalimumab; + D: plus DMARD. CZP: certolizumab; ETA: etanercept; GOL: golimumab; INF: infliximab; PBO: placebo; DMARD: disease-modifying anti-rheumatic drugs


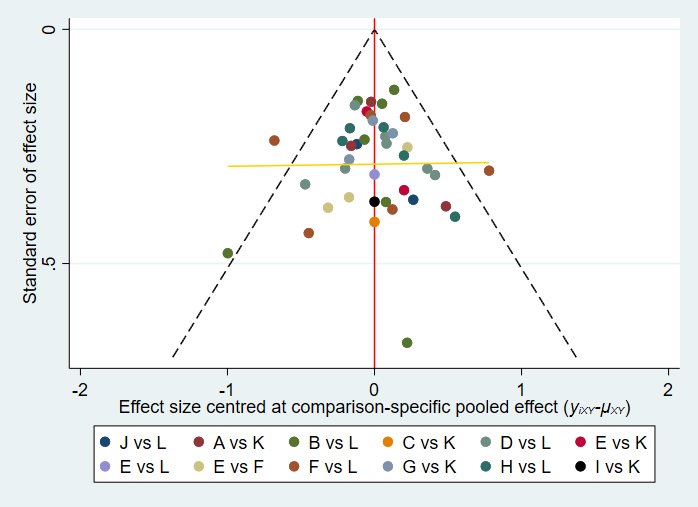


**Supplementary Figure 3** Network of funnel plot for infection. A: adalimumab; B: adalimumab + DMARD; C: certolizumab; D: certolizumab + DMARD; F: etanercept; G: etanercept + DMARD; H: golimumab; I: golimumab + DMARD; J: infliximab; K: infliximab + DMARD; L: DMARD; DMARD:disease-modifying anti-rheumatic drugs.


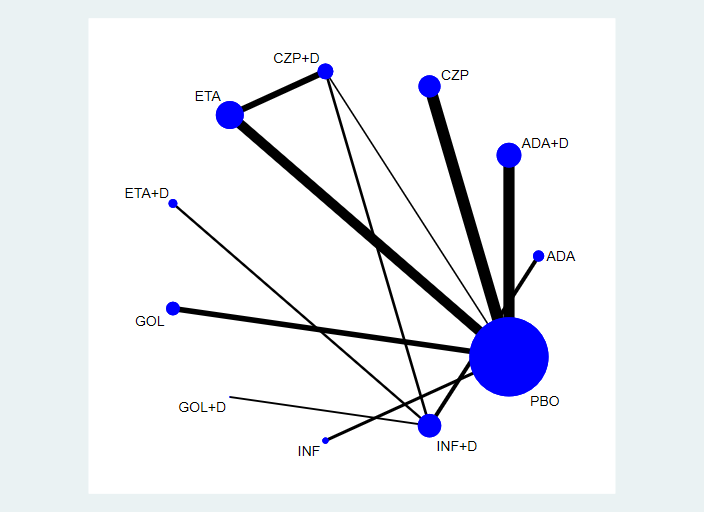


**Supplementary Figure 4** Network of treatment comparisons for serious infections. The size of the circles corresponds to the total number of people. Direct comparable treatments are connected with a line. ADA: adalimumab; + D: plus DMARD. CZP: certolizumab; ETA: etanercept; GOL: golimumab; INF: infliximab; PBO: placebo; DMARD: disease-modifying anti-rheumatic drugs.


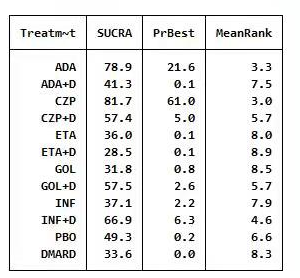


**Supplementary Figure 5** The analysis SUCRA of serious infections for 12 therapies. ADA: adalimumab; + D: plus DMARD. CZP: certolizumab; ETA: etanercept; GOL: golimumab; INF: infliximab; PBO: placebo; DMARD: disease-modifying anti-rheumatic drugs


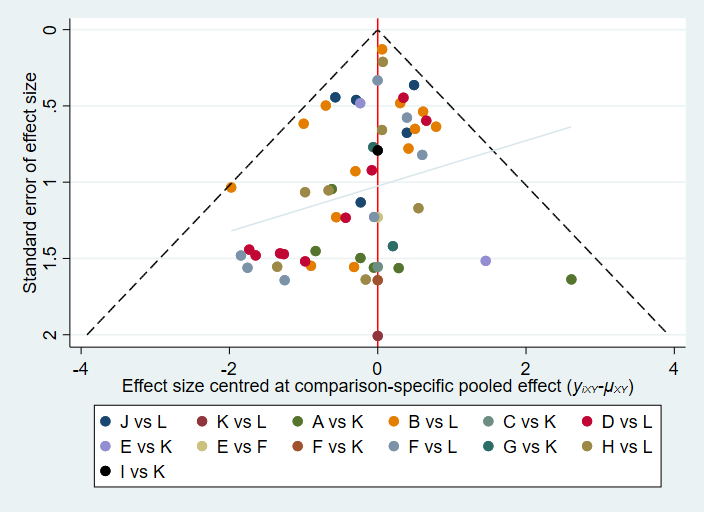


**Supplementary Figure 6** Network of funnel plot for serious infection. A: adalimumab; B: adalimumab + DMARD; C: certolizumab; D: certolizumab + DMARD; F: etanercept; G: etanercept + DMARD; H: golimumab; I: golimumab + DMARD; J: infliximab; K: infliximab + DMARD; L: DMARD; DMARD:disease-modifying anti-rheumatic drugs.


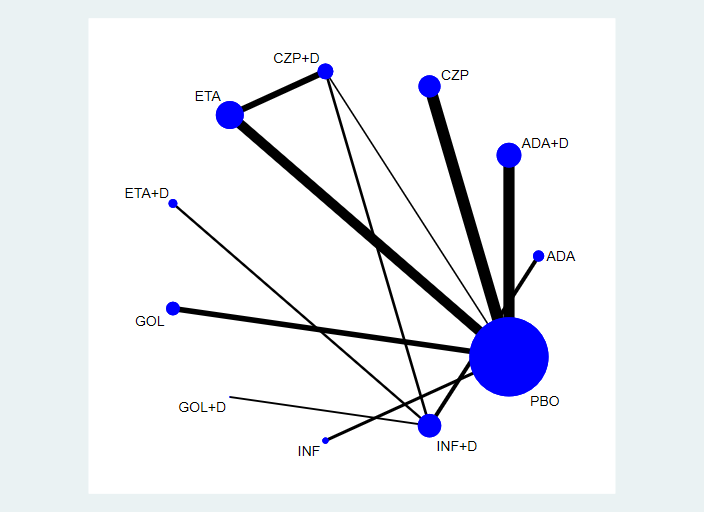


**Supplementary Figure 7** Network of treatment comparisons for malignant tumors. The size of the circles corresponds to the total number of people. Direct comparable treatments are connected with a line. ADA: adalimumab; + D: plus DMARD. CZP: certolizumab; ETA: etanercept; GOL: golimumab; INF: infliximab; PBO: placebo; DMARD: disease-modifying anti-rheumatic drugs


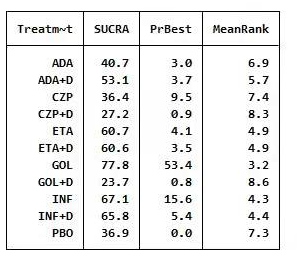


**Supplementary Figure 8** The analysis SUCRA of malignant tumors for 12 therapies. ADA: adalimumab; + D: plus DMARD. CZP: certolizumab; ETA: etanercept; GOL: golimumab; INF: infliximab; PBO: placebo; DMARD: disease-modifying anti-rheumatic drugs

## Supplementary Tables

| ADA | 0.84 (0.45,1.57) | 1.01 (0.42,2.44) | 0.95 (0.51,1.79) | 0.70 (0.44,1.10) | 0.80 (0.45,1.42) | 0.80 (0.54,1.20) | 0.72 (0.38,1.37) | 1.17 (0.52,2.61) | 1.42 (0.68,2.97) | 0.78 (0.59,1.04) | 0.70 (0.39,1.28) |
| --- | --- | --- | --- | --- | --- | --- | --- | --- | --- | --- | --- |
| 1.19 (0.64,2.22) | ADA+DMARD | 1.20 (0.44,3.28) | 1.13 (0.86,1.50) | 0.83 (0.54,1.28) | 0.95 (0.72,1.27) | 0.96 (0.51,1.79) | 0.86 (0.63,1.17) | 1.39 (0.55,3.54) | 1.69 (1.06,2.72) | 0.93 (0.53,1.62) | 0.84 (0.70,1.01) |
| 0.99 (0.41,2.39) | 0.83 (0.31,2.26) | CZP | 0.94 (0.34,2.58) | 0.69 (0.28,1.72) | 0.79 (0.30,2.10) | 0.80 (0.33,1.92) | 0.71 (0.26,1.98) | 1.15 (0.38,3.56) | 1.41 (0.48,4.14) | 0.77 (0.34,1.78) | 0.70 (0.26,1.87) |
| 1.05 (0.56,1.98) | 0.88 (0.67,1.17) | 1.06 (0.39,2.92) | CZP+DMARD | 0.74 (0.47,1.14) | 0.84 (0.62,1.13) | 0.85 (0.45,1.60) | 0.76 (0.55,1.04) | 1.23 (0.48,3.15) | 1.50 (0.93,2.41) | 0.82 (0.47,1.45) | 0.74 (0.60,0.91) |
| 1.43 (0.91,2.25) | 1.20 (0.78,1.84) | 1.44 (0.58,3.58) | 1.36 (0.87,2.11) | ETA | 1.14 (0.80,1.63) | 1.15 (0.73,1.81) | 1.03 (0.65,1.63) | 1.67 (0.73,3.83) | 2.03 (1.14,3.64) | 1.12 (0.78,1.59) | 1.01 (0.68,1.48) |
| 1.25 (0.70,2.22) | 1.05 (0.79,1.40) | 1.26 (0.48,3.35) | 1.19 (0.88,1.60) | 0.88 (0.61,1.25) | ETA+DMARD | 1.00 (0.56,1.79) | 0.90 (0.65,1.25) | 1.46 (0.59,3.61) | 1.78 (1.10,2.88) | 0.98 (0.59,1.61) | 0.88 (0.71,1.09) |
| 1.24 (0.83,1.87) | 1.05 (0.56,1.95) | 1.26 (0.52,3.04) | 1.18 (0.63,2.24) | 0.87 (0.55,1.37) | 1.00 (0.56,1.77) | GOF | 0.90 (0.47,1.72) | 1.45 (0.65,3.25) | 1.77 (0.84,3.72) | 0.97 (0.73,1.29) | 0.88 (0.48,1.60) |
| 1.39 (0.73,2.64) | 1.16 (0.86,1.58) | 1.40 (0.51,3.87) | 1.32 (0.96,1.80) | 0.97 (0.61,1.53) | 1.11 (0.80,1.53) | 1.11 (0.58,2.13) | GOF+DMARD | 1.62 (0.62,4.19) | 1.97 (1.20,3.23) | 1.08 (0.60,1.94) | 0.98 (0.77,1.24) |
| 0.86 (0.38,1.92) | 0.72 (0.28,1.83) | 0.87 (0.28,2.67) | 0.81 (0.32,2.09) | 0.60 (0.26,1.38) | 0.69 (0.28,1.69) | 0.69 (0.31,1.54) | 0.62 (0.24,1.60) | INF | 1.22 (0.44,3.37) | 0.67 (0.31,1.42) | 0.60 (0.24,1.51) |
| 0.70 (0.34,1.47) | 0.59 (0.37,0.95) | 0.71 (0.24,2.09) | 0.67 (0.41,1.08) | 0.49 (0.28,0.88) | 0.56 (0.35,0.91) | 0.56 (0.27,1.19) | 0.51 (0.31,0.83) | 0.82 (0.30,2.27) | INF+DMARD | 0.55 (0.28,1.09) | 0.49 (0.32,0.76) |
| 1.28 (0.96,1.71) | 1.08 (0.62,1.87) | 1.29 (0.56,2.98) | 1.22 (0.69,2.15) | 0.90 (0.63,1.28) | 1.02 (0.62,1.69) | 1.03 (0.77,1.37) | 0.92 (0.52,1.65) | 1.50 (0.70,3.18) | 1.82 (0.92,3.61) | PBO | 0.90 (0.53,1.53) |
| 1.42 (0.78,2.58) | 1.19 (0.99,1.44) | 1.44 (0.53,3.85) | 1.35 (1.10,1.66) | 0.99 (0.67,1.47) | 1.14 (0.91,1.41) | 1.14 (0.63,2.08) | 1.02 (0.81,1.30) | 1.66 (0.66,4.16) | 2.02 (1.31,3.11) | 1.11 (0.65,1.88) | DMARD |

**Supplementary Table 1** OR of infections for 12 therapies. Results below the diagonal are the rate ratios with 95% confidence intervals from the network meta-analysis of direct and indirect comparisons between the row-defining treatment and the column-defining treatment. Numbers in red highlight statistically significant results. ADA: adalimumab; + D: plus DMARD. CZP: certolizumab; ETA: etanercept; GOL: golimumab; INF: infliximab; PBO: placebo; DMARD: disease-modifying anti-rheumatic drugs

| **ADA** | 0.38 (0.04,3.88) | 2.10 (0.08,53.99) | 0.47 (0.04,5.17) | 0.32 (0.08,1.32) | 0.31 (0.03,3.07) | 0.26 (0.05,1.51) | 0.46 (0.04,4.86) | 0.30 (0.04,2.05) | 0.54 (0.05,5.69) | 0.42 (0.14,1.29) | 0.35 (0.03,3.61) |
| --- | --- | --- | --- | --- | --- | --- | --- | --- | --- | --- | --- |
| 2.65 (0.26,27.33) | **ADA+DMARD** | 5.56 (0.14,218.08) | 1.26 (0.69,2.30) | 0.84 (0.11,6.18) | 0.83 (0.49,1.44) | 0.70 (0.06,8.03) | 1.23 (0.80,1.88) | 0.80 (0.06,10.39) | 1.42 (0.88,2.31) | 1.11 (0.14,8.58) | 0.94 (0.76,1.16) |
| 0.48 (0.02,12.30) | 0.18 (0.00,7.06) | **CZP** | 0.23 (0.01,9.21) | 0.15 (0.01,3.60) | 0.15 (0.00,5.70) | 0.13 (0.00,3.51) | 0.22 (0.01,8.78) | 0.14 (0.00,4.39) | 0.26 (0.01,10.23) | 0.20 (0.01,4.21) | 0.17 (0.00,6.58) |
| 2.11 (0.19,23.05) | 0.80 (0.43,1.46) | 4.42 (0.11,180.11) | **CZP+DMARD** | 0.67 (0.08,5.26) | 0.66 (0.31,1.41) | 0.56 (0.05,6.76) | 0.98 (0.50,1.92) | 0.64 (0.05,8.72) | 1.13 (0.55,2.31) | 0.88 (0.11,7.29) | 0.75 (0.42,1.32) |
| 3.16 (0.76,13.18) | 1.19 (0.16,8.78) | 6.63 (0.28,158.03) | 1.50 (0.19,11.81) | **ETA** | 0.99 (0.14,6.88) | 0.84 (0.17,4.11) | 1.46 (0.19,11.03) | 0.95 (0.16,5.66) | 1.69 (0.22,12.94) | 1.33 (0.55,3.19) | 1.12 (0.15,8.15) |
| 3.18 (0.33,31.07) | 1.20 (0.70,2.06) | 6.66 (0.18,252.74) | 1.51 (0.71,3.20) | 1.01 (0.15,6.95) | **ETA+DMARD** | 0.84 (0.08,9.15) | 1.47 (0.79,2.74) | 0.96 (0.08,11.87) | 1.70 (0.88,3.30) | 1.33 (0.18,9.68) | 1.12 (0.68,1.85) |
| 3.78 (0.66,21.52) | 1.42 (0.12,16.27) | 7.91 (0.28,219.80) | 1.79 (0.15,21.63) | 1.19 (0.24,5.86) | 1.19 (0.11,12.92) | **GOL** | 1.75 (0.15,20.36) | 1.14 (0.15,8.76) | 2.02 (0.17,23.82) | 1.58 (0.42,5.97) | 1.34 (0.12,15.13) |
| 2.16 (0.21,22.69) | 0.81 (0.53,1.25) | 4.53 (0.11,179.81) | 1.02 (0.52,2.01) | 0.68 (0.09,5.15) | 0.68 (0.37,1.26) | 0.57 (0.05,6.66) | **GOL+DMARD** | 0.65 (0.05,8.61) | 1.16 (0.65,2.05) | 0.91 (0.11,7.14) | 0.76 (0.53,1.11) |
| 3.32 (0.49,22.60) | 1.25 (0.10,16.30) | 6.96 (0.23,212.88) | 1.57 (0.11,21.60) | 1.05 (0.18,6.25) | 1.05 (0.08,12.97) | 0.88 (0.11,6.78) | 1.54 (0.12,20.37) | **INF** | 1.78 (0.13,23.82) | 1.39 (0.30,6.57) | 1.18 (0.09,15.16) |
| 1.87 (0.18,19.82) | 0.70 (0.43,1.14) | 3.91 (0.10,156.44) | 0.88 (0.43,1.81) | 0.59 (0.08,4.51) | 0.59 (0.30,1.14) | 0.49 (0.04,5.82) | 0.86 (0.49,1.53) | 0.56 (0.04,7.51) | **INF+DMARD** | 0.78 (0.10,6.25) | 0.66 (0.43,1.02) |
| 2.39 (0.77,7.36) | 0.90 (0.12,6.94) | 5.00 (0.24,105.35) | 1.13 (0.14,9.32) | 0.75 (0.31,1.82) | 0.75 (0.10,5.45) | 0.63 (0.17,2.38) | 1.10 (0.14,8.72) | 0.72 (0.15,3.39) | 1.28 (0.16,10.22) | **PBO** | 0.84 (0.11,6.44) |
| 2.83 (0.28,28.84) | 1.07 (0.86,1.32) | 5.92 (0.15,230.99) | 1.34 (0.76,2.36) | 0.89 (0.12,6.52) | 0.89 (0.54,1.47) | 0.75 (0.07,8.48) | 1.31 (0.90,1.89) | 0.85 (0.07,10.97) | 1.51 (0.98,2.34) | 1.18 (0.16,9.04) | **DMARD** |

**Supplementary Table 2** OR of serious adverse events for 12 therapies.

Results below the diagonal are the rate ratios with 95% confidence intervals from the network meta-analysis of direct and indirect comparisons between the row-defining treatment and the column-defining treatment. Numbers in red highlight statistically significant results. ADA: adalimumab; + D: plus DMARD. CZP: certolizumab; ETA: etanercept; GOL: golimumab; INF: infliximab; PBO: placebo; DMARD: disease-modifying anti-rheumatic drugs

**Supplementary Table 3** OR of malignant tumors for 12 therapies.

Results below the diagonal are the rate ratios with 95% confidence intervalsfrom the network meta-analysis of direct and indirect comparisons between the row-defining treatment and the column-defining treatment. Numbers in red highlight statistically significant results. ADA: adalimumab; + D: plus DMARD. CZP: certolizumab; ETA: etanercept; GOL: golimumab; INF: infliximab; PBO: placebo; DMARD: disease-modifying anti-rheumatic drugs.

| **ADA** | 1.33 (0.10,18.34) | 0.68 (0.02,23.97) | 0.69 (0.04,11.16) | 1.74 (0.31,9.66) | 1.59 (0.15,16.86) | 5.12 (0.17,155.32) | 0.59 (0.03,10.93) | 2.06 (0.13,32.22) | 2.48 (0.14,43.40) | 2.01 (0.43,9.40) | 0.98 (0.08,12.10) |
| --- | --- | --- | --- | --- | --- | --- | --- | --- | --- | --- | --- |
| 0.75 (0.05,10.36) | **ADA+DMARD** | 0.51 (0.01,26.40) | 0.52 (0.13,2.10) | 1.31 (0.18,9.58) | 1.20 (0.38,3.77) | 3.85 (0.09,173.67) | 0.44 (0.09,2.30) | 1.55 (0.06,39.13) | 1.87 (0.40,8.75) | 1.51 (0.15,14.92) | 0.73 (0.35,1.53) |
| 1.47 (0.04,51.54) | 1.95 (0.04,100.50) | **CZP** | 1.01 (0.02,58.10) | 2.56 (0.09,76.89) | 2.34 (0.05,101.44) | 7.51 (0.09,624.07) | 0.87 (0.01,54.61) | 3.02 (0.06,154.06) | 3.64 (0.06,220.53) | 2.94 (0.12,72.62) | 1.43 (0.03,68.76) |
| 1.45 (0.09,23.43) | 1.93 (0.48,7.78) | 0.99 (0.02,56.69) | **CZP+DMARD** | 2.53 (0.28,22.69) | 2.31 (0.53,10.10) | 7.42 (0.15,374.26) | 0.86 (0.13,5.67) | 2.99 (0.10,85.98) | 3.60 (0.59,21.82) | 2.90 (0.24,34.47) | 1.41 (0.43,4.63) |
| 0.57 (0.10,3.18) | 0.76 (0.10,5.57) | 0.39 (0.01,11.76) | 0.40 (0.04,3.56) | **ETA** | 0.91 (0.18,4.63) | 2.94 (0.11,75.67) | 0.34 (0.03,3.60) | 1.18 (0.09,15.05) | 1.42 (0.14,14.10) | 1.15 (0.37,3.60) | 0.56 (0.09,3.55) |
| 0.63 (0.06,6.65) | 0.84 (0.27,2.63) | 0.43 (0.01,18.59) | 0.43 (0.10,1.90) | 1.09 (0.22,5.56) | **ETA+DMARD** | 3.21 (0.09,121.54) | 0.37 (0.07,2.06) | 1.29 (0.06,26.47) | 1.56 (0.31,7.86) | 1.26 (0.17,9.16) | 0.61 (0.25,1.48) |
| 0.20 (0.01,5.93) | 0.26 (0.01,11.73) | 0.13 (0.00,11.07) | 0.13 (0.00,6.80) | 0.34 (0.01,8.78) | 0.31 (0.01,11.76) | **GOL** | 0.12 (0.00,6.41) | 0.40 (0.01,17.97) | 0.49 (0.01,25.87) | 0.39 (0.02,8.21) | 0.19 (0.00,8.00) |
| 1.69 (0.09,31.22) | 2.25 (0.43,11.65) | 1.15 (0.02,72.56) | 1.17 (0.18,7.72) | 2.95 (0.28,31.25) | 2.69 (0.49,14.94) | 8.65 (0.16,480.41) | **GOL+DMARD** | 3.49 (0.11,112.08) | 4.20 (0.57,31.07) | 3.39 (0.25,46.66) | 1.65 (0.38,7.18) |
| 0.48 (0.03,7.58) | 0.65 (0.03,16.29) | 0.33 (0.01,16.85) | 0.33 (0.01,9.64) | 0.85 (0.07,10.76) | 0.77 (0.04,15.80) | 2.48 (0.06,110.79) | 0.29 (0.01,9.23) | **INF** | 1.20 (0.04,36.98) | 0.97 (0.10,9.45) | 0.47 (0.02,10.97) |
| 0.40 (0.02,7.03) | 0.54 (0.11,2.51) | 0.27 (0.00,16.62) | 0.28 (0.05,1.69) | 0.70 (0.07,6.95) | 0.64 (0.13,3.23) | 2.06 (0.04,109.92) | 0.24 (0.03,1.76) | 0.83 (0.03,25.48) | **INF+DMARD** | 0.81 (0.06,10.45) | 0.39 (0.10,1.53) |
| 0.50 (0.11,2.34) | 0.66 (0.07,6.57) | 0.34 (0.01,8.40) | 0.34 (0.03,4.09) | 0.87 (0.28,2.72) | 0.79 (0.11,5.78) | 2.55 (0.12,53.51) | 0.30 (0.02,4.06) | 1.03 (0.11,9.99) | 1.24 (0.10,16.03) | **PBO** | 0.49 (0.06,4.27) |
| 1.02 (0.08,12.71) | 1.36 (0.65,2.85) | 0.70 (0.01,33.58) | 0.71 (0.22,2.32) | 1.79 (0.28,11.33) | 1.63 (0.68,3.94) | 5.25 (0.12,220.36) | 0.61 (0.14,2.64) | 2.11 (0.09,49.00) | 2.55 (0.66,9.89) | 2.05 (0.23,18.01) | **DMARD** |
